# The ins and outs of RND efflux pumps in *Escherichia coli*

**DOI:** 10.3389/fmicb.2015.00587

**Published:** 2015-06-10

**Authors:** João Anes, Matthew P. McCusker, Séamus Fanning, Marta Martins

**Affiliations:** UCD Centre for Food Safety, School of Public Health, Physiotherapy and Population Science, UCD Centre for Molecular Innovation and Drug Discovery, University College DublinDublin, Ireland

**Keywords:** *Escherichia coli*, efflux pumps, antimicrobial resistance, antibiotics, biocides

## Abstract

Infectious diseases remain one of the principal causes of morbidity and mortality in the world. Relevant authorities including the WHO and CDC have expressed serious concern regarding the continued increase in the development of multidrug resistance among bacteria. They have also reaffirmed the urgent need for investment in the discovery and development of new antibiotics and therapeutic approaches to treat multidrug resistant (MDR) bacteria. The extensive use of antimicrobial compounds in diverse environments, including farming and healthcare, has been identified as one of the main causes for the emergence of MDR bacteria. Induced selective pressure has led bacteria to develop new strategies of defense against these chemicals. Bacteria can accomplish this by several mechanisms, including enzymatic inactivation of the target compound; decreased cell permeability; target protection and/or overproduction; altered target site/enzyme and increased efflux due to over-expression of efflux pumps. Efflux pumps can be specific for a single substrate or can confer resistance to multiple antimicrobials by facilitating the extrusion of a broad range of compounds including antibiotics, heavy metals, biocides and others, from the bacterial cell. To overcome antimicrobial resistance caused by active efflux, efforts are required to better understand the fundamentals of drug efflux mechanisms. There is also a need to elucidate how these mechanisms are regulated and how they respond upon exposure to antimicrobials. Understanding these will allow the development of combined therapies using efflux inhibitors together with antibiotics to act on Gram-negative bacteria, such as the emerging globally disseminated MDR pathogen *Escherichia coli* ST131 (O25:H4). This review will summarize the current knowledge on resistance-nodulation-cell division efflux mechanisms in *E. coli*, a bacteria responsible for community and hospital-acquired infections, as well as foodborne outbreaks worldwide.

## Introduction

*Escherichia coli* is a well-recognized human pathogen. While most strains do not cause disease, some serotypes are pathogenic. *E. coli* is the most common cause of UTIs worldwide, but can also cause bacteraemia and neonatal meningitis as well as serious food-borne infections. The recent emergence of specific serotypes such as O157:H7, responsible for food- and water-borne outbreaks in Europe (Money et al., 2010; Pennington, 2014) and the U.S. (Centers for Disease Control and Prevention [CDC], 2006), and the enterohaemorrhagic *E. coli* O104:H4 that caused the 2011 German outbreak, resulting in 53 deaths (Radosavljevic et al., 2014), pose a serious threat to public health. More recently, the worldwide pandemic clone *E. coli* O25:H4 ST131 has emerged harboring CTX-M-type beta-lactamases as well as several virulence genes that result in a MDR phenotype (Olesen et al., 2013).

Treatment of *E. coli* infections depends on the diagnosis. Antibiotic therapy normally involves the administration of co-trimoxazole, nitrofurantoin, or a fluoroquinolone and only in life-threatening situations a third-generation cephalosporin can be administrated (Piddock, 2006). The extensive use of fluoroquinolone-based antimicrobials, has been a major driver in the development of antibiotic resistant *E. coli* strains (Cagnacci et al., 2008; Lamikanra et al., 2011; Matsumura et al., 2013; Michael et al., 2014).

Antimicrobial resistance has been considered the new challenge of the 21st century (World Health Organization (WHO), 2014). The increased level of resistance to antimicrobial agents has raised serious questions concerning the way in which these therapeutic compounds are being used (Gilbert and McBain, 2001). Global organizations have expressed their concern on this issue, suggesting that increased focus and efforts are required to address this challenge (World Health Organization (WHO), 2014). The intensive use of antimicrobial compounds in the human clinical setting and in animals as growth promoters (Castanon, 2007) or as a preventive measure against infection, is considered to be one of the root causes for selection of resistant bacteria. The constant exposure to sub-lethal concentrations of antimicrobial compounds, along with commonly used biocides for disinfection processes, can play an important role in the selection and emergence of resistant strains (Andersson and Hughes, 2014; Capita et al., 2014). The use of certain antibiotics, specifically fluoroquinolones, has led to an increase in MDR phenotypes associated with the overexpression of efflux pumps (Wang et al., 2001). In addition, the presence of naturally occurring heavy metals and the use of chemicals in agriculture for fertilization of the soil can also induce the expression of efflux pumps in environmental strains leading to cross-resistance (Peltier et al., 2010). Strengthening our understanding of these resistance mechanisms will contribute to the development of new compounds that can ultimately help to overcome this challenge.

## Mechanisms of Antimicrobial Resistance

Gram-negative bacteria, like *E. coli*, have several mechanisms of resistance when it comes to surviving the selective pressure exerted by antimicrobial agents. Some mechanisms can be definitive whereas others may reverse when the selective pressure is released or completely removed. Resistance can occur due to: (i) accumulation of mutations involved in specific antimicrobial targets (e.g., mutations in quinolone resistance-determining regions (QRDRs) in *gyrA, gyrB, parE*, and *parC* genes) (Moon et al., 2010); (ii) antimicrobial inactivation/modification (e.g., production of β-lactamase enzymes; Poole, 2002); (iii) acquisition of mobile genetic elements such as plasmids, transposons, or integrons acquired by HGT (Carraro et al., 2014; Gillings, 2014); (iv) alteration in the cell wall composition (e.g., lipopolysaccharide modification; Gunn, 2001); (v) reduced expression of cell wall porins, resulting in decreased influx of antimicrobials (Masi and Pagès, 2013); and (vi) over-expression of efflux pumps (Wang et al., 2001).

## Efflux Pumps

Classicaly, efflux pumps can be classified into five different families: the ABC superfamily; the major facilitator superfamily (MFS); the MATE family; the SMR family and the RND family (Poole, 2007; Li and Nikaido, 2009; Delmar et al., 2014). Recently, the proteobacterial antimicrobial compound efflux (PACE) family was identified in some Gram-negative bacteria. However, *E. coli* strains do not seem to encode PACE efflux proteins unless carried by mobile genetic elements (Hassan et al., 2015a,b). While all the efflux pump families are well distributed among Gram-negative bacteria, RND are responsible for the extrusion of a broad range of compounds (Nikaido and Pagès, 2012). Depending on the efflux mechanism different energy sources can be used. ABC transporters for instance, use ATP as the energy source for extrusion of toxic compounds (Lubelski et al., 2007), whereas MATE pumps are driven by Na^+^/H^+^ drug antiport systems (Alvarez-Ortega et al., 2013). The MFS, SMR, and RND pumps are PMF driven, which means that these are dependent on the pH gradient. For instance, *E. coli* has been shown to be able to extrude ethidium bromide more efficiently at lower pH values when compared to higher pH values (Martins et al., 2009; Amaral et al., 2011).

## RND General Structure and Substrates

Resistance-nodulation-cell division transporters operate as part of a tripartite system composed of the RND pump located in the inner membrane, a periplasmic adaptor protein from the MFP family and an OMP belonging to the outer membrane factor (OMF) family located in the outer membrane (Nikaido, 2011). The OMP TolC, for example, works in combination with other RND, ABC, and MFS efflux pumps (Tanabe et al., 2009; Turlin et al., 2014). The absence of any of these components renders the entire complex non-functional. Nonetheless, the efflux systems show a cooperative interaction between them and can act sequentially when one fails (Lee et al., 2000; Tal and Schuldiner, 2009). RND transporters can be classified into two different subfamilies according to their substrates, the hydrophobic and amphiphilic efflux RND (HAE-RND) family and the heavy metal efflux RND (HME-RND) family (Nies, 2003). In *E. coli* there are five efflux transporters that belong to the HAE-RND subfamily, AcrAB (Tikhonova and Zgurskaya, 2004; Pos, 2009), AcrAD (Rosenberg et al., 2000; Elkins and Nikaido, 2002), AcrEF (Nishino and Yamaguchi, 2001), MdtAB (Baranova and Nikaido, 2002; Nagakubo et al., 2002), and MdtEF (Kobayashi et al., 2006; Zhang et al., 2011). In contrast, there is only one efflux transporter that belongs to the HME-RND, the CusCFBA (Su et al., 2011a; Chacón et al., 2014; Delmar et al., 2014).

## Acriflavine (Acr) Efflux System

**FIGURE 1:**
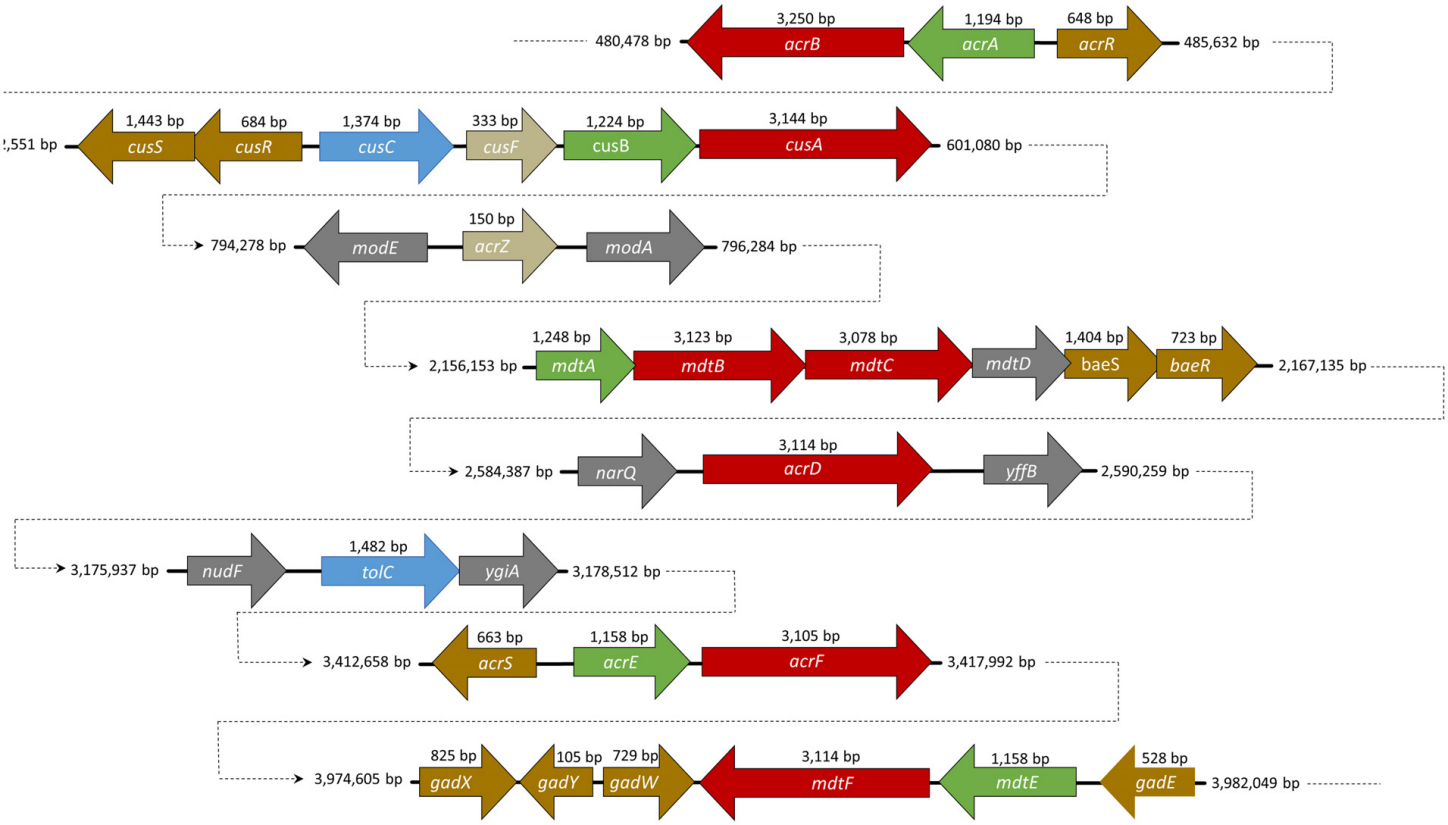
**A schematic representation of the position and size of the resistance-nodulation-cell division (RND) efflux pump genes in the genome of *Escherichia coli* str. K-12 substr. W3110**. RND genes are described in red, membrane fusion protein (MFP) in green, and the outer membrane protein (OMP) in blue. Small proteins such as AcrZ and CusF are indicated in beige. Regulators are marked with the color yellow.

AcrAB is encoded in a single operon for the RND and the MFP, under the control of the transcriptional repressor, AcrR and AcrS (Wang et al., 2001; Hirakawa et al., 2008; **Figure 1**). SdiA (Wei et al., 2001) and CpxRA (Yang et al., 2014) have also been shown to regulate expression of *acrAB*. The outer membrane component, TolC, is coded elsewhere on the chromosome (Fralick, 1996) and is part of the *marA*/*soxS*/*rob* regulon (Warner and Levy, 2010).

In the last years, there has been more evidence favoring a stoichiometry of 3:6:3, comprising an AcrB trimer, an AcrA hexamer and a TolC trimer (Tikhonova et al., 2011; Xu et al., 2011; Du et al., 2014). However, the functional stoichiometry remains unclear and under some controversy (Zgurskaya and Nikaido, 2000; Fernandez-Recio et al., 2004; Symmons et al., 2009).

**FIGURE 2:**
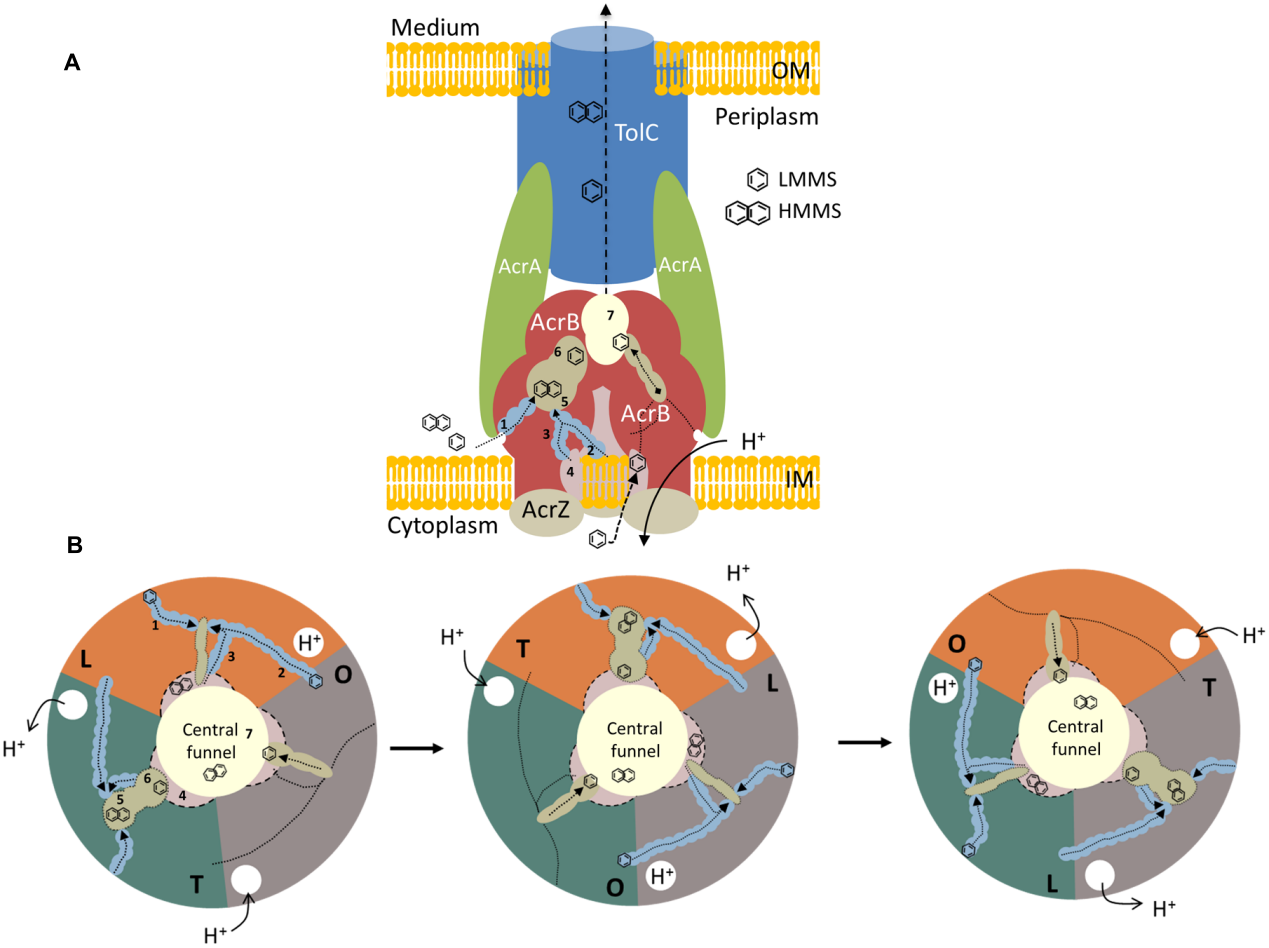
**A model representation of AcrABZ-TolC efflux pump. (A)** Side view of a schematic model of a tripartite efflux system in Gram-negative bacteria. RND protein AcrB is described in red, the MFP, AcrA is indicated in green, and the OMP, TolC in blue. The small protein AcrZ is colored in beige. OM, outer membrane; IM, inner membrane. **(B)** Top view of the different stages of the ligand extrusion mechanism in AcrB RND efflux pump. Each color represents a protomer of the AcrB protein. 1: lower external depression or cleft channel; 2: vestibule channel; 3: central cavity channel; 4: central cavity; 5: proximal binding pocket; 6: distal binding pocket; 7: central funnel. LMMS, low molecular mass substrates; HMMS, high molecular mass substrates. Adapted from (Murakami et al., 2002, 2006; Seeger et al., 2006; Husain et al., 2011; Nakashima et al., 2011).

The RND protein AcrB is composed of 1,049 amino acids and is distributed throughout the transmembrane domain and the large periplasmic domain (Husain and Nikaido, 2010; **Figure 2A**). The first symmetrical crystal structure for AcrB protein was resolved by Murakami et al. (2002) at a 3.5 Å resolution in which three AcrB protomers were organized as a homotrimer. Co-crystallization of AcrB with several ligands (including 6-rhodamine 6G, ethidium, dequalinium and ciprofloxacin) showed that these ligands bind near the transmembrane domain and in various positions within the binding pocket. The binding is established primarily through hydrophobic, aromatic stacking, and van der Waals interactions (Yu et al., 2003). Asymmetric crystal structures of AcrB were later resolved using minocycline, doxorubicin (Murakami et al., 2006), ethidium, dequalinium (Seeger et al., 2006), and designed ankyrin repeat proteins (DARPins), the latter being an inhibitor designed specifically for AcrB, as a substrate (Sennhauser et al., 2007).

The asymmetric structure obtained indicated that each protomer takes on a different conformation, which is related to a different stage of the ligand binding and extrusion mechanism (**Figure 2B**). One of the monomers presents a loose (L) conformation. Here, the substrates can enter from three different open channels (**Figures 2A,B**; 1, 2, and 3). Substrates can enter *via* the lower external depression or cleft (**Figures 2A,B**; 1) in the periplasmic side close to the outer leaflet away from the membrane surface. Substrates can also enter through the vestibule, located between the protomers (**Figures 2A,B**; 2), facing toward the periplasm, close to the external surface of the membrane bilayer (Murakami et al., 2006; Sennhauser et al., 2007). The last channel (**Figures 2A,B**; 3) in which substrates can enter is located directly in the central cavity (**Figures 2A,B**; 4; Husain and Nikaido, 2010; Husain et al., 2011). The central cavity is involved in the transport of substrates from the cytosol (Yu et al., 2003). Once the substrate that derives from the cytoplasm or the periplasm binds to one of the channels, the binding pocket expands to accommodate the substrate. Once expanded, the substrate moves through the uptake channel, or tunnel, binding to the different locations within the multisite binding pocket (Murakami et al., 2006). The binding step occurs in the interior of the periplasmic domain at the tight protomer (T). High molecular mass substrates (HMMS), such as rifampicin or erythromycin, bind to the proximal binding pocket (**Figures 2A,B**; 5; Nakashima et al., 2011). Low molecular mass substrates (LMMS), as minocycline or doxorubicin, on the other hand, travel through the proximal pocket and bind further up on the distal pocket (**Figures 2A,B**; 6; Murakami et al., 2006). The extrusion of the substrate is then dependent on the last open protomer (O) of the AcrB trimer. Here, the conformation of the central helix of the protomer is changed due to the protonation closing the open channels and opening the gate into the central funnel (**Figures 2A,B**; 7). Finally, the substrate is pushed out to the central funnel by the shrinking of the binding pocket where it will bind to the TolC domain and is subsequently extruded from the bacterial cell (Murakami et al., 2006; Seeger et al., 2006; Sennhauser et al., 2007; Nikaido and Pagès, 2012). An in depth review on the structure and transport of AcrB can be found elsewhere (Yu et al., 2003; Pos, 2009; Husain and Nikaido, 2010; Nakashima et al., 2011; Li et al., 2015).

The MFP AcrA (**Figure 2A**) is anchored to the inner membrane by the N-terminal lipid modification. This protein is composed of a membrane proximal (MP) domain, a β-barrel domain, a lipoyl domain and an α-hairpin domain (Symmons et al., 2009). The later domain is known to interact with the outer membrane TolC whereas the first three domains are responsible for the interaction with AcrB (Elkins and Nikaido, 2003; Lobedanz et al., 2007; Kim et al., 2010a). Other studies show that AcrA is able to bind both RND and OMP proteins independently with nanomolar affinity (Tikhonova et al., 2011). AcrA oligomerisation and pH proved to be essential for AcrB affinity (Tikhonova et al., 2009, 2011; Xu et al., 2011; Kim et al., 2010a, 2015). However, some thermodynamic interaction studies have shown that AcrA-TolC binds spontaneously whereas the interaction between AcrB and TolC is not thermodynamically favored (Touzé et al., 2004). AcrA function as a dimeric unit and each AcrA dimer has a propensity to form a trimer when in contact with an AcrB protomer, establishing a AcrA–AcrB stoichiometry of 6:3 (Tikhonova et al., 2011; Xu et al., 2011). The AcrA MP domain is also known to play an important role in the assembly of the tripartite efflux system (Ge et al., 2009; Weeks et al., 2010; Tikhonova et al., 2011).

The TolC outer membrane channel is made up of three TolC protomers that form a cannon shaped tunnel of 140 Å (Koronakis et al., 2000; **Figure 2A**). This channel makes the connection between the periplasmic space and the cell exterior through the outer membrane (Hinchliffe et al., 2013). The 12-stranded β-barrel, formed by four β-strands from each TolC protomer, are constitutively open to the external medium, whereas the periplasmic α-barrel comprises 12 α-helices in an antiparallel arrangement, four from each protomer (Koronakis et al., 2000). Here an aspartate ring of residues establish a constriction that effectively closes the TolC pore to substrates (Higgins et al., 2004). TolC assumes a closed conformation when inactive. Only a small portion of TolC is used to support efflux activity and it is expressed at lower levels. The expression of AcrAB has no effect on TolC expression (Krishnamoorthy et al., 2013). The TolC protein is also part of others efflux systems such as the EmrAB-TolC or the MacAB-TolC of the MFS and ABC superfamilies, respectively, (Tanabe et al., 2009; Lu and Zgurskaya, 2012).

The assembly mechanism of the tripartite system is not fully understood, to date. Therefore, several models have been generated over the years. The *Adaptor Wrapping Model*, assumes that AcrB and TolC connect individually; plasmon surface resonance data also confirms this interaction (Tikhonova et al., 2011). TolC binds to the AcrB crown through the outer helices H3 and H4 (Weeks et al., 2010). As a result of this interaction, the inner helices H7 and H8 relax exposing the intraprotomer groove. The exposed intraprotomer groove is a high-affinity site for AcrA (Bavro et al., 2008). Binding to the exposed grooves of TolC cause alterations in the MP domain of AcrA (Krishnamoorthy et al., 2013); these conformational changes induce the opening of the TolC (Bavro et al., 2008). However, the partial opening of TolC, using a TolC mutant, rendered the tripartite system unstable and lead to disassembly of AcrAB-TolC efflux pump (Tikhonova et al., 2011). To date, there seems to be no consensus as to how the opening of TolC actually occurs.

The other model generated over the last years is the *Adaptor Bridging Model*. This model assumes no direct interaction between AcrB and TolC (Kim et al., 2010a; Xu et al., 2012; Du et al., 2014). Thermodynamic data has also shown that this interaction is not favored (Touzé et al., 2004). This model was generated based on the structure of other efflux systems with similar tripartite composition to AcrAB-TolC (Yum et al., 2009; Xu et al., 2012). These models also claim the fact that cross-linking interaction with cysteine only indicates that the protein are in close proximity with each other; it does not mean that they are stable (Touzé et al., 2004). In fact, attempts at cross-linking have shown that the proteins do not form functional complexes (Kim et al., 2010a; Xu et al., 2011), or barely functional complexes (Du et al., 2014). In this model, AcrB and TolC are bridged in the periplasm through an AcrA hexamer (Xu et al., 2011; Du et al., 2014; Kim et al., 2015). When packed, AcrA protomers generate a funnel-like structure with a sealed central channel, closed to the periplasm (Du et al., 2014). The AcrA α-helical hairpins interact with the periplasmic ends of the α-helical coiled coils of TolC in an intermeshing-like cogwheel structure (Xu et al., 2011; Kim et al., 2015). The β-barrel and lipoyl domains of AcrA form a triangular hollow chamber, who’s bottom opens into the funnel of AcrB (Xu et al., 2011; Du et al., 2014). The interaction with the α-helical hairpins of AcrA change the conformation of the TolC hexamer to the open form (Du et al., 2014). TolC remains in this open form throughout the entire transport cycle.

Recently, a small protein AcrZ, formerly designated YbhT, was shown to interact with AcrB through the inner membrane (Hobbs et al., 2012). The *acrZ* gene is co-regulated by MarA, Rob and SoxS, the same regulators of AcrAB efflux pump. The gene is located between two molybdenum-flanking operons involved in molybdenum uptake and the regulation of molybdenum cofactor synthesis (see **Figure 1**). Structures such as tetracycline or acriflavine, or other substrates with several polycyclic features, are suggestive of the molybdenum cofactor. Enzymes such as xanthine dehydrogenase and aldehyde oxidoreductase (that requires a molybdenum cofactor) detoxify compounds that may be extruded through AcrB RND pump (Hobbs et al., 2012). The AcrZ protein was shown to help AcrAB-TolC complex to recognize and export chloramphenicol, tetracycline and puromycin (Hobbs et al., 2012). These authors suggested that AcrZ might trigger some conformational changes in the periplasmic domain affecting the recognition and capture of substrates with lower hydrophobicity.

AcrAB-TolC efflux system is known to be responsible for the extrusion of a broad range of compounds such as lipophilic antimicrobial drugs, i.e., penicillin G, cloxacillin, nafcillin, macrolides, novobiocin, linezolid, and fusidic acid (Nikaido, 2011); antibiotics (such as fluoroquinolones, cephalosporins, tetracyclines; Nikaido and Takatsuka, 2009); various dyes (i.e., crystal violet, acridine, acriflavine, ethidium, 6-rhodamine 6G); detergents [sodium dodecyl sulfate (SDS) and Triton X-100; Pos, 2009]; organic solvents (hexane, cyclohexane); steroid hormones (bile acids, estradiol, and progesterone) (Elkins and Mullis, 2006); essential oils (Fadli et al., 2014); and others (see **Table 1**). A common feature of all the substrates is that, to some extent, all contain lipophilic domains (Nikaido, 1996). However, the way these substrates interact with the phospholipid bilayer and the RND pumps is not yet completely defined.

**Table 1:** Resistance-nodulation-cell division (RND) efflux pumps substrates and regulators.

Efflux pump	Substrates	RND Regulator(s)	Reference
AcrAB-TolC	β-lactams, quinolones, tetracyclines, tigecycline, chloramphenicol, steroid hormones, lincosamides, benzene, cyclohexane, SDS, Triton X-100, rifampicin, bile salts, free fatty acids, geraniol, enterobactin, triclosan, chlorhexidine, quaternary ammonium compounds, acriflavin, ethidium bromide	AcrR AcrS SdiA MarRAB Rob SoxS CpxRA	Perreten et al. (2001), Wang et al. (2001), Rahmati et al. (2002), Rosenberg et al. (2003), Hirata et al. (2004), Elkins and Mullis (2006), Poole (2007), Hirakawa et al. (2008), Lennen et al. (2013), Shah et al. (2013), Horiyama and Nishino (2014)
AcrAD-TolC	Aminoglycosides, steroid hormones, enterobactin, β-lactams, quinolones, Sodium dodecyl sulfate (SDS), deoxycholate	BaeR SdiA CpxRA	Nishino and Yamaguchi (2001), Wei et al. (2001), Hirakawa et al. (2003), Elkins and Mullis (2006), Poole (2007), Nishino et al. (2010), Horiyama and Nishino (2014), Kobayashi et al. (2014)
AcrEF-TolC	Quinolones, tigecycline, solvents	H-NS SdiA	Jellen-Ritter and Kern (2001), Kobayashi et al. (2001), Wei et al. (2001), Hirata et al. (2004), Nishino and Yamaguchi (2004)
MdtABC-TolC	Novobiocin, bile salts, enterobactin, quinolones, fosfomycin, benzalkonium, SDS, zinc, myricetin	BaeSR CpxRA	Nishino and Yamaguchi (2001), Baranova and Nikaido (2002), Nagakubo et al. (2002), Hirakawa et al. (2003), Nishino et al. (2010), Kim and Nikaido (2012), Wang and Fierke (2013), Horiyama and Nishino (2014)
MdtEF-TolC	Erythromycin, doxorubicin, benzalkonium, SDS, deoxycholate, crystal violet, ethidium bromide, nitrosyl indole, rhodamine 6G, tetraphenylphosphonium bromide, free fatty acids	H-NS YdeO CRP GadX ArcBA GadE EvgAS RpoS DsrA	Nishino and Yamaguchi (2001, 2002, 2004), Kobayashi et al. (2006), Nishino et al. (2008a,b, 2009, 2011), Zhang et al. (2011), Deng et al. (2013), Lennen et al. (2013)
CusCFBA	Silver, copper, fosfomycin, ethionamide, dinitrobenzene	CusSR	Nishino and Yamaguchi (2001), Conroy et al. (2010), Kim et al. (2011), Gudipaty et al. (2012)

AcrD, another acriflavine RND-type efflux pump, plays a similar role to AcrB. In contrast with the other acriflavine resistance efflux systems, *acrD* does not form an operon with the MFP gene *acrA* (**Figure 1**; Rosenberg et al., 2000). This gene is under the control of the regulons BaeR (Hirakawa et al., 2003), SdiA (Wei et al., 2001), and CpxRA (Nishino et al., 2010). With 1,037 amino acids and 66.1% homology with AcrB, AcrAD-TolC has a different substrate specificity mainly for hydrophilic substrates such as aminoglycosides (Rosenberg et al., 2000), negatively charged β-lactams (Kobayashi et al., 2014) and mild specificity for SDS, deoxycholate, novobiocin, cholic acid (Nishino and Yamaguchi, 2001), progesterone (Elkins and Mullis, 2006), and others. AcrD was used as a model to understand how RND pumps capture their substrates. This efflux pump was shown to capture aminoglycosides from both the periplasm and the cytoplasm (Aires and Nikaido, 2005). The results were then extrapolated in part to AcrB due to the close homology between both proteins. A chimeric study between AcrB and AcrD showed that the replacement of the two large external loops of AcrD with the equivalent loops of AcrB altered the substrate range of AcrD to a broader one, more typical of AcrB. Conversely, the replacement of the two large loops in AcrB with those of AcrD lead to an efflux pump that had a narrower substrate range, similar to that of an AcrD efflux pump. This demonstrated the importance these large periplasmic loops of AcrB and AcrD play in the substrate range of these pumps (Elkins and Nikaido, 2002).

AcrEF-TolC is also part of the acriflavine efflux system. The efflux system is encoded in an operon wherein the RND *acrF* gene is located together with the MFP gene *acrE*, with both genes under the control of the regulator *sdiA* (Wei et al., 2001). Histone-like proteins (e.g., H-NS, HU, IHF) are a key component of the bacterial nucleoid and play an important role in global gene regulation (Nishino and Yamaguchi, 2004). The inactivation of H-NS increases the expression of AcrEF, indicating that H-NS represses the expression of AcrEF. The RND protein AcrF has 1,034 amino acids and 77.6% homology with AcrB, whereas AcrE has 385 amino acids and 69.3% homology with AcrA. Due to the close homology observed it is predicted that this system assembles in a similar manner as AcrAB.

The complex, AcrEF is postulated to be functionally identical to AcrAB due to its similar broad substrate range (Nishino and Yamaguchi, 2001). Nonetheless, *acrEF* expression is very low under laboratory conditions (Lau and Zgurskaya, 2005). Deletion *acrEF* produced no observable effect on the resistance phenotype in *E. coli* (Sulavik et al., 2001). Currently, is still unclear under what physiologic conditions AcrEF is expressed.

The recombination of insertion sequence (IS) elements upstream of the *acrEF* operon increases the expression of the efflux system (Misra et al., 2015). Using an *acrB* mutant with increased expression of AcrEF the hypersentivity to solvents generated by the mutant was supressed (Kobayashi et al., 2001); the same pattern was also seen for fluoroquinolones (Jellen-Ritter and Kern, 2001).

The acriflavine efflux system is one of the most studied systems and presents a broad range of substrate resistance. Understanding how these tripartite systems act in different conditions has given us some insights that could be used to design and develop new inhibitor compounds.

## Multidrug Transport (Mdt) Efflux System

MdtABC-TolC is encoded in a single operon under the control of the two-component regulatory system, BaeSR (**Figure 1**; Nagakubo et al., 2002). During envelope stress this efflux system can also be activated by the CpxRA regulon (Nishino et al., 2010). The *mdtD* gene, which is also part of this operon, codes for a MFS transporter (Baranova and Nikaido, 2002). Nonetheless, this protein is not necessary for increased resistance to antimicrobials. Recently, MdtD has been associated with efflux of iron and citrate, being renamed as IceT (Frawley et al., 2013). MdtABC-TolC efflux system is responsible for the extrusion of substrates such as novobiocin, bile salts (Nagakubo et al., 2002), quinolones, fosfomycin, detergents (Nishino and Yamaguchi, 2001), zinc (Wang and Fierke, 2013), and myricetin (Kim and Nikaido, 2012).

The MdtABC-TolC efflux system presents a heterotrimeric conformation for the RND pump, in contrast to the pattern seen for AcrB, AcrD, AcrF, and CusA. The RND pump MdtBC, formerly known as YegNO, is composed by two MdtB protomers of 1,040 amino acids each, and one MdtC protomer with 1,025 residues (Kim et al., 2010b). Remarkably, these two protomers only show 49% similarity between them (Kim and Nikaido, 2012). In this case, each protomer has a specific function; MdtC is likely to be involved in substrate binding and extrusion whereas MdtB has been shown to induce conformational changes in the trimer, when the substrate binds to MdtC, leading to proton translocation (Kim and Nikaido, 2012). There is little structural information about this efflux system as there is no crystallographic data available to date. A homology model based on the AcrB crystal structure showed that MdtC has a broad tunnel beginning at the external cleft and continuing all the way to the interior edge of the binding pocket. The tunnel appears to narrow before it reaches the binding pocket (Kim and Nikaido, 2012). Based on this model, a substrate mechanism was predicted in which the substrate binds first to the peripheral site near the cleft. The binding of the substrate may induce proton translocation through the MdtB subunit(s), which will cause conformational changes mainly at the opening of the tunnel and the expansion of the binding pocket in the MdtC protomer, in a manner similar to what is observed in AcrAB (Kim and Nikaido, 2012).

MdtEF-TolC is the other known multidrug efflux system in this family, formerly referred to as YhiUV-TolC. The genes encoding this efflux system are located in an operon (**Figure 1**) under the control of *gadX* (Nishino et al., 2008b), *gadY* (Kobayashi et al., 2006) and *gadE* (Deng et al., 2013), which are regulators of acid resistance (**Table 1**). This operon can be activated in complex cascades or directly by several major regulators, such as EvgAS (Nishino and Yamaguchi, 2002); ArcBA (Deng et al., 2013); RpoS (Kobayashi et al., 2006); YedO (Nishino et al., 2009); or by the MdtEF repressor of cyclic AMP receptor protein (Nishino et al., 2008a), or the histone-like protein H-NS (Nishino and Yamaguchi, 2004). MdtEF efflux pump function is related with cell growth, its maximal expression level is achieved in late stationary phase of *E. coli* growth (Kobayashi et al., 2006). Proteins MdtE and MdtF share 55 and 71% homology with AcrA and AcrB, respectively. Due to this fact the same conformational structure was assumed for the purpose of this review. MdtEF is known to induce resistance to oxacillin, cloxacillin, nafcillin, erythromycin, rhodamine 6G, and SDS under the controlled expression of the regulator, GadX (Nishino et al., 2008b). It can also induce resistance to benzalkonium, deoxycholate (Nishino and Yamaguchi, 2001), indole (Zhang et al., 2011), and others. In *E. coli*, MdtEF has an important role in cell growth under anaerobic conditions (Zhang et al., 2011). MdtEF mutants have shown susceptibility to indole nitrosative derivatives, a by-product formed when the bacterium metabolizes nitrate under anaerobic conditions. It was also observed that under respiratory stress MdtEF was able to extrude erythromycin (Zhang et al., 2011).

## Copper Transporting (Cus) Efflux System

The CusCFBA system is the only HME-RND identified in *E. coli* to date. It is responsible for the extrusion of silver (Ag^+^) and copper (Cu^+^). Although recognized for the extrusion of these two ions it was also found to induce resistance to fosfomycin (Nishino and Yamaguchi, 2001), dinitrophenol, dinitrobenzene, and ethionamide (Conroy et al., 2010). The *cus* genes, *cusCFBA*, are all located in the same operon (**Figure 1**) under the control of *cusR* and *cusS* encoding a response regulator and a histidine kinase, respectively, (Gudipaty et al., 2012). This efflux system plays an important role in Cu^+^ homeostasis in bacterial cells (Rademacher and Masepohl, 2012). The Cus system, like all the RND efflux pumps presents a tripartite structure or a tetrapartite structure, if CusF is taken into consideration. For the purpose of this review, the system will be considered as a tripartite system, as was the case for the AcrAB-TolC system and AcrZ.

The system is composed by the RND efflux pump (CusA); the MFP (CusB); and by the OMP (CusC). These proteins assemble together in a stoichiometry identical to the AcrB, CusA_(3)_:CusB_(6)_:CusC_(3)_ (Su et al., 2011b; Delmar et al., 2014).

With 1,047 amino acids CusA was first resolved at 3.5 Å suggesting a homotrimer configuration. Each protomer is composed by 12 transmembrane α-helices, four of each protruding into the cytoplasm and two into the periplasm (Long et al., 2010). Consequently, Cu^+^ and Ag^+^ were found to bind to the residues M573, M623, and M672 located at the bottom of the periplasmic cleft together with other conserved residues (Su et al., 2012). Once bound, both Cu^+^ and Ag^+^ appeared to induce significant conformational changes in both the periplasmic and transmembrane domains of CusA (Long et al., 2010; Su et al., 2012). The changes seemed to create a doorway for metal ions to enter the periplasmic domain of the pump. The cleft, which is initially closed, is open in the presence of Ag^+^ or Cu^+^ revealing the binding site. At the same time the changes caused by the binding may also relate to transmembrane signaling, which are thought to initiate proton translocation across the membrane (Delmar et al., 2013).

Four distinct methionine pairs have been identified on the inside of the channel formed by each protomer of the CusA pump. Three of these pairs: M410–M501, M403–M486, and M391–M1009 are found below the binding site in the transmembrane domain, and one, M271–M755, is located at the bottom of the periplasmic funnel. This channel, together with the three-methionine residues from the binding site, spans the entire length of each protomer. This represents a pathway for the transport of metal ions (Su et al., 2011a). Metal ions can enter through the binding site inside the cleft directly through the periplasmic cleft or *via* the cytoplasm through the methionine pairs within the transmembrane region. The metal ions bound at the methionine pair in the periplasmic funnel are released into the central funnel following the methionine pathway (Su et al., 2011a).

The second component of the Cus efflux system is the periplasmic membrane protein CusB. With 407 amino acids, it was first resolved at 3.4 Å (Su et al., 2009). Based on the crystal structure, it was initially thought to form a dimer. Subsequently, it has been shown that CusB forms a hexameric channel directly above the periplasmic domain of CusA (Su et al., 2011b; Long et al., 2012). The structure of the CusB hexamer mimics an inverted funnel and follows the same stoichiometry as AcrA, AcrB_(3)_:AcrA_(6)_:TolC_(3)_.

Four domains, three β-domains and one α-helical domain, compose each protomer of CusB. The first β-domain is located above the outer-leaflet of the inner membrane and it interacts directly with the CusA through the periplasmic domain (Su et al., 2011a). Both Ag^+^ and Cu^+^ bind to the CusB domain-1 at the residue positions M324, F358, and R368. The hexameric CusB channel is primarily created by β-barrels in the lower half, whereas the upper half is an entirely α-helical tunnel. The inner surface of the channel is predominantly negatively charged, which suggests a capacity to bind positively charged metal ions (Delmar et al., 2014). Methionine residues M21, M36, and M38 of CusB are most likely Cu^+^ and Ag^+^ binding sites (Bagai et al., 2007). These residues are located outside the periplasmic cleft of CusA, the same cleft that was shown to harbor the three-methionine binding sites. Therefore, CusB may transfer the bound metal ions at this location into the periplasmic cleft of CusA for extrusion (Su et al., 2011b).

CusF is a small chaperone protein of 110 amino acids and was first crystallized in 2005 at 1.5 Å resolution (Loftin et al., 2005). CusF exhibits a small five-stranded β-barrel composed of two antiparallel, three-stranded β-sheets packed orthogonally. Three conserved residues H36, M47, M49, and W44 located in β-strands 2 and 3, have been shown to interact with Cu^+^ and Ag^+^ (Loftin et al., 2007; Xue et al., 2008). Native mass spectrometry has shown that the N-terminal region of CusB can acquire Cu^+^ from CusF (Mealman et al., 2012). It was proposed that under anaerobic conditions, due to the accumulation of Cu^+^ and Ag^+^ in the periplasm, there is a strong up-regulation of the metal chaperone CusF and that the former would work as a scavenger of metal ions and help fill all the available CusB binding sites (Kim et al., 2011; Chacón et al., 2014). The proposed model also suggests that CusB activates and deactivates the CusA pump depending on the ion levels in the periplasmic space. CusF interaction with CusB appears to have an activating effect in this cascade, initiated by the opening of the periplasmic cleft.

The OMP of the Cus system, CusC, with 460 amino acids, was first crystallized in 2011 at a resolution of 2.3 Å (Kulathila et al., 2011). CusC assembles as a homotrimer creating a large cylindrical channel, the largest found in the outer membrane family. Structurally, the cannon shape is identical to TolC and OprM, however, CusC is believed to work only in the Cus efflux system (Delmar et al., 2014). The interior surface of the channel is highly electronegative allowing for the binding of positively charge ligands. The transmembrane domains appear to be open at the bottom entrance, whereas it appears to be closed at the far end by van der Waals interactions. Each protomer contains a tri-acylated N-terminal cysteine residue shown to be covalently linked to the outer membrane *via* a thioester bond (Kulathila et al., 2011; Delmar et al., 2013). In a docking model, CusC appears to interact with CusAB at the interior of the upper half of the channel formed by the α-helical domain of CusB, primarily through coiled-coil interaction (Long et al., 2012).

The Cus system is well characterized for Cu^+^ and Ag^+^, nonetheless, the mechanism of action for other substrates needs to be further characterized.

## Functions of RND Efflux Pumps in *E. coli*

During growth, *E. coli* produces secondary metabolites that can be toxic to the cell. There are three ways of eliminating these metabolites. This can be achieved by inclusion of these metabolites into a second metabolic pathway; enzymatic degradation to non-toxic metabolites or extrusion of metabolites generated to the external environment. RND efflux pumps can also contribute to eliminate secondary metabolites. In a study using deleted genes for enterobactin biosynthesis (*entA* and *entE*), gluconeogenesis (*glpX*), cysteine biosynthesis (*cysH*), and purine biosynthesis (*purA*), the results showed no or diminished effect on the AcrAB promoter in an AcrB mutant strain (Ruiz and Levy, 2014). This suggests that this efflux pump is able to extrude compounds that are toxic or have a signaling role. In *E. coli*, the secretion of iron-chelating siderophore enterobactin from the periplasm is associated with AcrAB, AcrAD, and MdtABC RND efflux pumps (Horiyama and Nishino, 2014). Using a triple mutant *acrB*, *acrD*, and *mdtABC* a significant reduction in the extrusion of enterobactin from the cells was noted. Secretion of metabolites is therefore important for the proper development of the cell. Most of these metabolites regulate master regulators such as MarA or SoxS (Amaral et al., 2011). Therefore, based on these observations the sensor dosage hypothesis was developed. This hypothesis contemplates a homeostatic loop in which the intercellular concentration of metabolites reaches a certain threshold, at which they activate transcriptional regulators such as MarA, SoxS, and Rob (Rosner and Martin, 2009; Amaral et al., 2011). These regulators activate their target genes encoding proteins that function in the extrusion mechanism. When the basal levels of these metabolites are restored, the expression of the transcriptional regulators is restored also.

Considering the human host, in a food-borne infection scenario, *E. coli* has to transit through the human gastrointestinal (GI) tract in order to successfully colonize the intestine. To accomplish this it must survive pH variations and toxicity of the different environments. The presence of bile acids poses an additional barrier to bacterial survival. Bile acids are released into the duodenum and are present along the GI tract, affecting the cell membrane, and DNA of *E. coli* (Merritt and Donaldson, 2009). Bile salts are known to induce the expression of AcrAB-TolC in *E. coli* (Thanassi et al., 1997; Rosenberg et al., 2003) allowing the bacteria to survive in such environments. Other mammalian steroid hormones that are released in bile, also activate AcrB, AcrD, and MdtF RND pumps (Elkins and Mullis, 2006). Recent studies described the effect that bile salts have on alerting (signaling) *E. coli* for the entrance into the small intestine. In a transcriptomic study designed to further explore the effect of bile salts on *E. coli* O157:H7, these were found to induce significant changes in the activation of genes associated with iron scavenging and metabolism, thereby, preparing the bacteria to survive in an iron limiting environment (Hamner et al., 2013).

AcrD and AcrF RND efflux pumps were reported to be up-regulated when bacteria are in the sessile state, i.e., biofilm state. Inhibition of these pumps led to a decrease in biofilm formation caused by *E. coli*-causing UTI strains (Kvist et al., 2008). Some extracellular components of the *E. coli* biofilm matrix, such as cellulose, are hydrophilic (Beloin et al., 2008). As it is known AcrD have specificity for these substrates. AcrD mutants have shown to be unable to form biofilms (Matsumura et al., 2011); these results illustrate the importance of RND efflux pumps in the colonization processes.

Cell-to-cell communication plays an important role in bacterial stress and cell organization. *In vitro* assays with MDR *E. coli* using lomefloxacin and ceftazidime showed over-expression of *marA* and *sdiA* (Tavío et al., 2010). SdiA is believed to behave as a quorum sensor in *E. coli* (Shimada et al., 2014) that controls the expression of AcrAB (Rahmati et al., 2002). Although *E. coli* does not have *N*-acylhomoserine lactone system, these bacteria can detect quorum sensing signals secreted by other organisms. Understanding how RND efflux pumps interact with quorum sensing mechanisms is important for future development of new compounds capable of inhibiting biofilm structures and interfering with bacterial cell-to-cell communication.

## “More In and Less Out” – How to Overcome Resistance Using Inhibitors of RND Pumps

Once the structure of the RND efflux pumps was solved and the assembling mechanism determined it was possible to start the rational development of new compounds capable of inhibiting their function. This could be achieved due to the fact that all the RND efflux systems work in the same way, changing only their substrates. However, to our knowledge, there are no compounds capable of inhibiting all RND efflux pumps and that could be used at the present time in clinical practice.

The use of so-called EPIs in combination with antibiotics (designated as adjuvant therapy) has shown some interesting results in overcoming the innate and induced resistance of bacteria due to efflux (Bohnert and Kern, 2005). The adjuvant therapy can be tested by combining the action of an EPI with an antibiotic or a substrate of a given RND efflux pump at sub-inhibitory concentrations and measuring its efflux rate (Coldham et al., 2010). RND efflux pumps can be inhibited by different ways; for example by interfering with the PMF mechanism or by inhibiting/competing with the binding site of the RND pump.

Inhibition of the PMF can be achieved using a PMF uncoupler such as carbonyl cyanide *m*-chlorophenylhydrazone (CCCP; Viveiros et al., 2005; Ikonomidis et al., 2008). This compound is mainly used for the screening of RND efflux pumps activity. Other compound, Phenylalanine-Arginine-β-naphthylamide (PAβN) was identified as an EPI by assaying an array of compounds against *Pseudomonas aeruginosa* (Lomovskaya et al., 2001). A few years later, PAβN was described as being not an inhibitor of RND efflux pumps, but rather a competitor (Viveiros et al., 2008). Recently, new data has shown that PAβN acts as an inhibitor of AcrAB and AcrEF efflux systems when used in low concentrations. At higher concentrations this compound showed not only inhibitory activity toward the mentioned efflux pumps but also an effect in destabilizing the cell wall membrane (Misra et al., 2015). PAβN was also found to bind to the upper portion of the central cavity and also the periplasmic domain to one side of the deep external cleft (Nikaido and Takatsuka, 2009). PAβN is known to be a successful compound in reverting the resistance to tetracycline (Viveiros et al., 2005) and to macrolides when compared to other known EPIs (Kern et al., 2006).

In *E. coli*, compounds such as PAβN, chloramphenicol and others have been shown to improve the efflux of cephalosporins (Kinana et al., 2013). This effect could be due to the fact that cephalosporins bind to a different site in the large binding pocket of the RND pump.

Naphthylpiperazines are also one type of RND inhibitor that could be used to target efflux pumps. Specifically, 1-(1-naphthylmethyl)-piperazine (NMP) is known to have an inhibitory effect on efflux pumps. When used in combination with fluoroquinolones this compound also showed a reduction in fluoroquinolone resistance in *E. coli* (Bohnert and Kern, 2005; Kern et al., 2006). A similar result was obtained when MDR *E. coli* of animal origin was assessed wherein NMP was able to reverse resistance to ciprofloxacin, tetracycline and florfenicol. However, NMP showed to be inefficient in reversing resistance to ampicillin (Marchetti et al., 2012).

The use of phenothiazines in combination with antibiotics has also being studied for potential reversal of bacterial resistance. Chlorpromazine (CPZ) and thioridazine (TZ), known antipsychotic drugs (Amaral and Molnar, 2012), have been shown to be active against *E. coli* (Viveiros et al., 2005). These compounds also showed to be able to reduce the resistance to aminoglycosides (Coutinho et al., 2010) as well as the capacity to be used for plasmid curing (Radhakrishnan et al., 1999; Spengler et al., 2003). Other recent compounds that should be considered include pimozide (Bohnert et al., 2013) or the specifically designed MBX2319 (Opperman et al., 2014; Vargiu et al., 2014) that also proved to have a reducing effect in the resistance to some antibiotic classes; a similar effect to the one seen by the previously described EPIs.

Based on the studies described above it seems that the use of different EPIs conducts to different results depending on the antibiotic used in the combination tested. These differences are possibly due to the different binding sites of each antibiotic/EPI. Classification of the different EPIs based on their activity in the pump or in combination with the different antimicrobial classes could provide essential information for future research.

## Final Conclusion

The extensive use of antimicrobials has resulted in an increased pressure being exerted in different ecological niches. Data collected so far allow us to assume that the resistant profile seen is not only due to the extensive use of antimicrobials but also due to the innate capacity of the bacteria to extrude a broad range of substrates. It is therefore important to characterize efflux pumps and their mechanism of action, in different bacteria. Understanding how efflux mechanisms can contribute to quorum sensing is of paramount importance for the development of new molecules that can target key genes or proteins in this cascade. Regarding RND efflux pumps in *E. coli* the crystal structure of several pumps such as the MdtABC is crucial for the elucidation of the binding pockets composition as well as their substrates for these types of pumps.

In the future, the combined use of EPIs with antibiotics appears to be a promising therapy to be applied in the fight against antibiotic resistance. Therefore, the development of new inhibitor molecules based on the models generated by the crystal structure of the efflux pumps will allow performing a more target-specific inhibition.

## Conflict of Interest Statement

The authors declare that the research was conducted in the absence of any commercial or financial relationships that could be construed as a potential conflict of interest.

## Abbreviations

ABC: ATP-binding cassette
EPI: efflux pump inhibitor
HGT: horizontal gene transfer
MATE: multidrug and toxic compound extrusion
MDR: multidrug resistant
MFP: membrane fusion protein
OMP: outer membrane protein
PMF: proton-motive force
RND: resistance-nodulation-cell division
SMR: small multidrug resistance
UTI: urinary tract infection.
